# Type 1 tyrosinemia in Finland: a nationwide study

**DOI:** 10.1186/s13023-020-01547-w

**Published:** 2020-10-12

**Authors:** Linnea Äärelä, Pauliina Hiltunen, Tea Soini, Nina Vuorela, Heini Huhtala, Pasi I. Nevalainen, Markku Heikinheimo, Laura Kivelä, Kalle Kurppa

**Affiliations:** 1 Center for Child Health Research, Tampere University, Tampere, Finland; 2 Department of Pediatrics, Tampere University Hospital, Tampere, Finland; 3 Children’s Hospital and Pediatric Research Center, University of Helsinki and Helsinki University Hospital, Helsinki, Finland; 4 Faculty of Social Sciences, Tampere University, Tampere, Finland; 5 Department of Internal Medicine, Tampere University Hospital, Tampere, Finland; 6 Department of Pediatrics, Seinäjoki Central Hospital and the University Consortium of Seinäjoki, Seinäjoki, Finland; 7 Tampere Center for Child Health Research, Arvo Building, Arvo Ylpön katu 34, 33520 Tampere, Finland

**Keywords:** Tyrosinemia, Succinylacetone, Liver transplant, Nitisinone, Screening

## Abstract

**Background:**

Introduction of nitisinone and newborn screening (NBS) have transformed the treatment of type 1 tyrosinemia, but the effects of these changes on the long-term outcomes remain obscure. Also, the predictors for later complications, the significance of drug levels and the normalization of laboratory and imaging findings are poorly known. We investigated these issues in a nationwide study.

**Results:**

Type 1 tyrosinemia was diagnosed in 22 children in 1978–2019 in Finland. Incidence was 1/90,102, with a significant enrichment in South Ostrobothnia (1/9990). Median age at diagnosis was 5 (range 0.5–36) months, 55% were girls and 13 had homozygotic Trp262X mutation. Four patients were detected through screening and 18 clinically, their main findings being liver failure (50% vs. 100%, respectively, p = 0.026), ascites (0% vs. 53%, p = 0.104), renal tubulopathy (0% vs. 65%, p = 0.035), rickets (25% vs. 65%, p = 0.272), growth failure (0% vs. 66%, p = 0.029), thrombocytopenia (25% vs. 88%, p = 0.028) and anaemia (0% vs. 47%, p = 0.131). One patient was treated with diet, seven with transplantation and 14 with nitisinone. Three late-diagnosed (6–33 months) nitisinone treated patients needed transplantation later. Kidney dysfunction (86% vs. 7%, p = 0.001), hypertension (57% vs. 7%, p = 0.025) and osteopenia/osteoporosis (71% vs. 14%, p = 0.017) were more frequent in transplanted than nitisinone-treated patients. Blood/serum alpha-fetoprotein decreased rapidly on nitisinone in all but one patient, who later developed intrahepatic hepatocellular carcinoma. Liver values normalized in 31 months and other laboratory values except thrombocytopenia within 18 months. Imaging findings normalized in 3–56 months excluding five patients with liver or splenic abnormalities. Low mean nitisinone concentration was associated with higher risk of severe complications (r = 0.758, p = 0.003) despite undetectable urine succinylacetone.

**Conclusions:**

Prognosis of type 1 tyrosinemia has improved in the era of nitisinone, and NBS seems to provide further benefits. Nevertheless, the long-term risk for complications remains, particularly in the case of late diagnosis and/or insufficient nitisinone levels.

## Background

Tyrosinemia is an autosomal recessively inherited metabolic disease presenting with three clinically distinct subtypes. The majority of patients have type I tyrosinemia (TT1, OMIM 276,700), types II and III being extremely rare [1–3]. The global incidence is ~ 1/100,000–1/200,000, but TT1 has enriched in certain areas, particularly in Northern Europe and Canada [4–7]. The disease is caused by a homozygous or compound heterozygous mutation in the gene on chromosome 15q25, leading to a lack of fumarylacetoacetate hydrolase (EC 3.7.1.2) and an ensuing accumulation of blood tyrosine, succinylacetoacetate and succinylacetone (SA) [6, 8, 9]. Typical presentation includes liver failure and kidney tubular dysfunction, other reported findings being, for example, growth failure, rickets and pseudo-porphyric crises [6, 9–12]. Untreated TT1 increases the risk for liver carcinoma [12, 13].

Liver transplantation was the only cure for TT1 until the discovery of nitisinone [14]. Although the drug is effective in preventing the production of toxic metabolites, the accumulation of tyrosine must be prevented by protein restriction [11]. Furthermore, although data is scarce, nitisinone-treated patients may also develop complications [6, 15, 16], particularly in the case of delayed diagnosis or persistently high tyrosine levels [6]. However, optimal long-term concentrations of plasma nitisinone and tyrosine are unclear. Newborn screening (NBS) has improved the short-term outcomes of TT1 patients [16, 17], but long-term results are still lacking [12]. Altogether, natural history studies of TT1 in the era of nitisinone and NBS are scant and have concentrated on a few geographical areas [16].

In Finland, the treatment of TT1 is centralized, with systematically maintained medical records and NBS programmes launched in 2014. This has enabled us to evaluate the incidence, changing features and long-term outcomes of TT1 in a nationwide setting.

## Results

### Incidence, family background and genetics

**Fig. 1:**
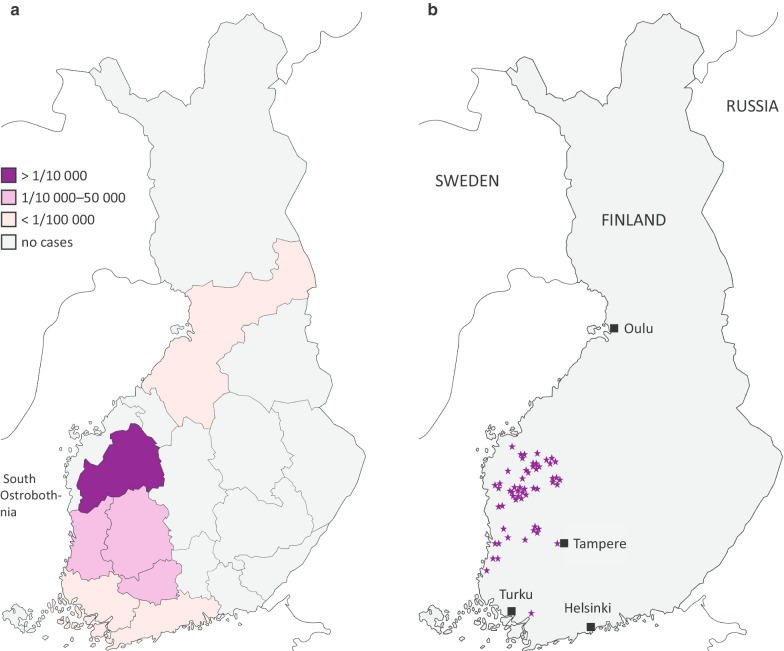
Incidence of type 1 tyrosinemia in 19 Finnish provinces (**a**) and the family origins of the patients (**b**)

Twenty-one patients were diagnosed in the period 1987–2018, and one in 1978. Additional search resulted in > 2000 metabolic/liver patients, none of whom had TT1. Incidence was 1/90,102 with significant enrichment in South Ostrobothnia (Fig. 1a), the area from which most patients originated (Fig. 1b). Two patients were of non-Finnish origin, and one of these had consanguineous parents. Three families had a history of TT1. Thirteen patients had a homozygous Finnish type c.786G > A, (p.Trp262X) mutation. Four patients had a compound heterozygous mutation, including Trp262X + c.1062 + 5G > A (p.?), Trp262X + unidentified other mutation, c.205del (p.Ser69fs) + c.554-1G > T (p.?) and c.191delA (p.Gln64fs) + c.191delA (p.Gln64fs). The latter two patients were originally from Kosovo and Iraq.

### Pregnancy and neonatal data

No pregnancy complications were reported. Median births weight was 0.1 (− 1.4 to 2.2) SD, length 0.4 (range − 1.3 to 3.8) SD, and head circumference 0.1 (− 1.3 to 1.8) SD. Of note, glucose value was measured in 20 out of the 22 newborns, and 45% of them needed intensified surveillance due to hypoglycaemia and 30% due to transient hypotonia. Five hypoglycaemic patients needed oral glucose supplementation and four intravenous infusion for a few days. Three of those with hypoglycaemia received TT1 diagnosis by NBS. Glucose value is measured frequently from Finnish newborns before discharging them from hospital.

### Characteristics at diagnosis

Twelve (55%) patients were girls and the median age was 5 (range 0.5–36) months. Eighteen patients were found clinically, the main findings including acute abdomen (n = 8), septicaemia (n = 4), ascites (n = 3), rickets (n = 2) and intestinal bleeding (n = 1). Three were found by NBS and one by family screening. They had fewer clinical (Table 1), laboratory (Table 2) and imaging abnormalities (Table 3) and higher calcium and prothrombin time (PT) and lower conjugated bilirubin levels (Table 2) than those detected clinically. One patient had kidney dysfunction (increased plasma creatinine and urea, oliguria) while 11 had tubulopathy (acidosis and abnormal urine protein and/or glucose and/or microglobulin). Median height was − 1.3 (− 1.8 to 0.1) SD in screened and − 1.7 (− 3.1 to 0.7) SD in clinically-detected patients (p > 0.05) respectively. Three clinically-detected children had bilateral and two unilateral inguinal hernia and two also scrotum hernia; they all had ascites.

**Table 1:** Clinical findings at diagnosis in clinically-detected and screen-detected patients with type 1 tyrosinemia

	Clinically-detected, n = 18		Screen-detected, n = 4		P value
	n	%	n	%	
*Symptoms*					
Fever	9	50.0	0	0	0.115
Recurrent vomiting	7	38.9	0	0	0.263
Melena/haematochezia	5	27.8	0	0	0.535
Diarrhoea	4	22.2	0	0	0.554
*Clinical findings*					
Liver failure	18	100.0	2	50.0	0.026
Growth failure	12	66.6	0	0	0.029
Kidney tubulopathy	11^a^	64.7	0	0	0.035
Jaundice	1	5.6	0	0	0.999
*Laboratory findings*					
Thrombocytopenia	15^a^	88.2	1	25.0	0.028
Metabolic acidosis	12^b^	75.0	1	25.0	0.101
Hypoglycaemia	9	50.0	2	50.0	0.999
Anaemia	8^a^	47.1	0	0	0.131
No symptoms or findings	0	0	1	25.0	0.182

^a^Data missing for 1 patient

^b^Data missing for 2 patients

**Table 2:** Laboratory findings at diagnosis in clinically-detected and screen-detected patients with type 1 tyrosinemia

	Clinically-detected, n = 18			Screen-detected, n = 4			P value
	n^a^	Median	Range	n^a^	Median	Range	
Age, months	18	6	2–36	4	1	0–31	0.098
AFP, kU/l	16	148,725	5990–420,800	4	82,868	6470–487,300	0.682
ALT, U/l	17	45	15–111	4	32	10–63	0.362
Calcium, mmol/l	15	2.15	1.26–2.70	4	2.60	2.34–2.60	0.014
Creatinine, μmol/l	14	25	12–103	4	27	24–32	0.327
DBil, μmol/l	12	17	6–38	4	5	3–11	0.013
γ-GT, U/l	14	136	43–328	4	99	91–134	0.327
NH4+ , μmol/l	15	74	36–141	4	65	38–87	0.530
Phosphate, mmol/l	16	0.86	0.22–2.64	4	1.85	0.77–2.19	0.064
PT, %	16	11	0–37	4	35	15–68	0.029
TBil, μmol/l	17	30	16–48	4	61	14–106	0.144
Tyrosine, μmol/l	16	384	100–840	4	490	452–734	0.099

*AFP* alpha-fetoprotein, *ALT* alanine aminotransferase, *DBil* conjugated bilirubin, *γ-GT* γ-glutamyl transferase, *NH4+* ammonium ion, *PT*, prothrombin time, *TBil* total bilirubin

^a^Data available

**Table 3:** Radiological findings at diagnosis in clinically-detected and screen-detected patients with type 1 tyrosinemia

	Clinically-detected, n = 17		Screen-detected, n = 4		P value
	n	%	n	%	
Hepatic nodules	17	100	2	50.0	0.029
Rickets	11	64.7	1	25.0	0.272
Hepatomegaly	10	58.8	1	25.0	0.311
Renomegaly	10	58.8	1	25.0	0.311
Ascites	9	52.9	0	0	0.104
Splenomegaly	6	35.3	0	0	0.281
CNS findings	3^a^	21.4	0	0	0.999
Cardiac findings	2^b^	13.3	0	0	0.999
No findings	0	0	1	25.0	0.190

The conducted imaging studies included wrist X-ray, abdominal, cardiac and cranial ultrasound and liver and brain magnetic resonance imaging or computer tomography. Cardiac ultrasound was available for 19 patients, Central nervous system (CNS) imaging was done for 16 patients and the other imaging studies for all 21 patients

^a^Two cases with resolving cerebral atrophy and one craniopharyngioma

^b^One mild mitral regurgitation and one atrium septum defect

### Initial treatment and short-term outcomes

One patient was treated with diet and seven with transplantation. One explant contained hepatocellular carcinoma, six cirrhosis and four cell atypia. Fourteen patients started nitisinone (median 1.0, range 0.9 –1.3 mg/kg/day). Eleven of them had dietary challenges and two needed temporary gastrostomy. Median hospitalization time, including the time before and after diagnosis and during liver transplantation, was 150 (range 78–195) days for children before the nitisinone era and 17 (2–45) days for children on nitisinone (p < 0.001).

### Long-term outcomes

Median follow-up time of all patients was 16 (range 3–32) years and 12 (3–24) years on nitisinone. One patient died (at the age of 7 years) before the era of transplantation, one (29 years) with transplant due to unknown cause and one (15 years) later transplanted patient for intracerebral haemorrhage. Median age of the surviving patients was 12 (3–24) years on nitisinone and 30 (29–32) with initial liver transplant.

Kidney dysfunction, hypertension and reduced bone mineral density were more common in transplanted than among nitisinone-treated patients, but not after adjusting for current age (Table 4). Four patients needed liver re-transplant(s) and one kidney transplant due to severe kidney dysfunction after liver transplantation, and four patients had surgical complications. Three late-diagnosed (6–33 months) patients on nitisinone also needed transplant due to elevation of alpha-fetoprotein (AFP) and new/persisting nodules in imaging studies raising suspicion of intrahepatic malignancy. One of them had non*-*metastatic hepatocellular carcinoma and two had cirrhosis. One nitisinone-treated patient had pseudo-porphyric crisis while having undetectable urine SA but low serum nitisinone (13 µmol/l). None had eye complications. Three screen-detected patients also had subsequent health problems (developmental delay, learning difficulties, osteopenia). Median adult height was − 1.4 (− 0.5 to − 3.9) SD in nitisinone-treated and − 1.5 (0.2 to − 2.0) SD in transplanted patients (p = 0.639).

**Table 4:** Long-term complications in tyrosinemia patients treated primarily either with liver transplantation or with nitisinone medication

	Transplantation, n = 7		Nitisinone, n = 14		P value
	n	%	n	%	
Kidney dysfunction^a^	6	85.7	1^b^	7.1	0.001^e^
Hypertension^c^	4	57.1	1	7.1	0.025^f^
Osteopenia/osteoporosis	5	71.4	2	14.3	0.017 ^g^
Osteoporotic fractures	2	28.6	0	0.0	0.100
Growth failure	2	28.6	3	21.4	0.999
Learning difficulties	4	57.1	4	28.6	0.346
Neurological symptoms^d^	2	28.6	2	14.3	0.440
Developmental delay	1	14.3	2	14.3	0.999
Any complication	7	100	8	57.1	0.061 ^h^

^a^One patient needed kidney transplant

^b^The patient had received liver transplant before the development of kidney dysfunction

^c^All patients with hypertension had received liver transplant and two had secondary cardiac hypertrophy

^d^Two patients had seizures, one had seizure in childhood and porphyrin crises at the age of 13 years, and one had facial paresis

^e−h^P = 0.999 for each if adjusted for current age. Kidney dysfunction and hypertension appeared at the age of 14–25 years, osteoporosis/osteopenia and fractures at the age of 6–20 years, neurological symptoms/developmental delay at the age of 3–17 years and growth failure at the age of 1–3 years

### Predictors for long-term complications

Males had more often ≥ 2 complications (100% vs. 50%, p = 0.015), whereas there was no association between complications and age or clinical presentation at diagnosis. Those with multiple complications had lower median calcium (r = −0.447, p = 0.055) and higher creatinine (r = 0.496, p = 0.036), alkaline phosphatase (ALP, r = 0.483, p = 0.027) and gamma-glutamyl transferase (γ-GT, r = 0.547, p = 0.019) levels at diagnosis, and lower nitisinone levels during follow-up (r = −0.758, p = 0.003). Growth delay at diagnosis also predicted later growth disturbances (p = 0.045).

### Normalization of the laboratory and imaging results

**Fig. 2:**
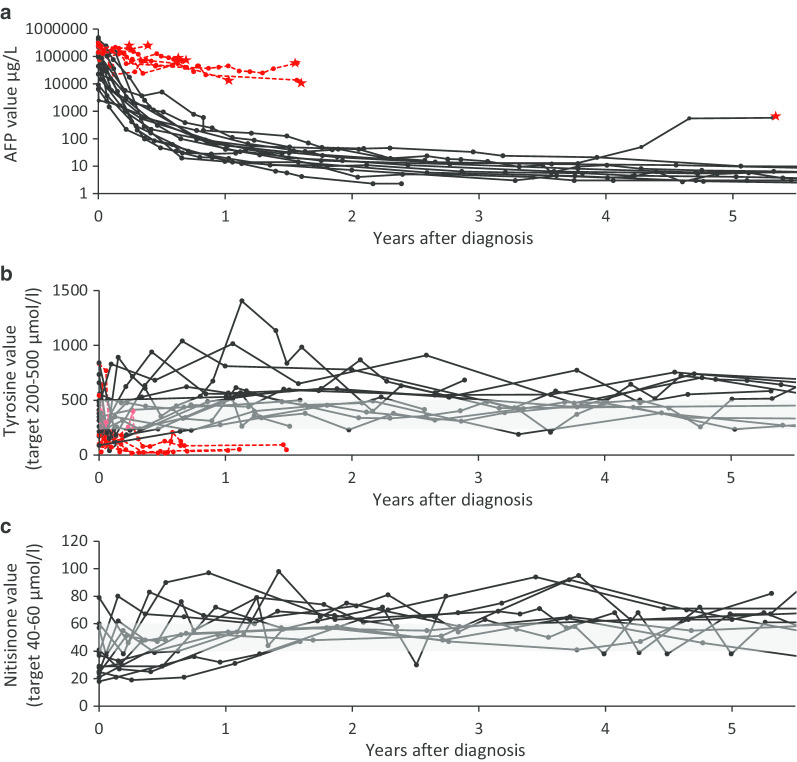
Individual blood alpha-fetoprotein (**a**), tyrosine (**b**) and nitisinone (**c**) values of the study patients. Black lines denote nitisinone-treated patients and red lines the values of the liver transplanted patients from diagnosis until the transplantation (star). Grey area denotes the recommended target range

**Fig. 3:**
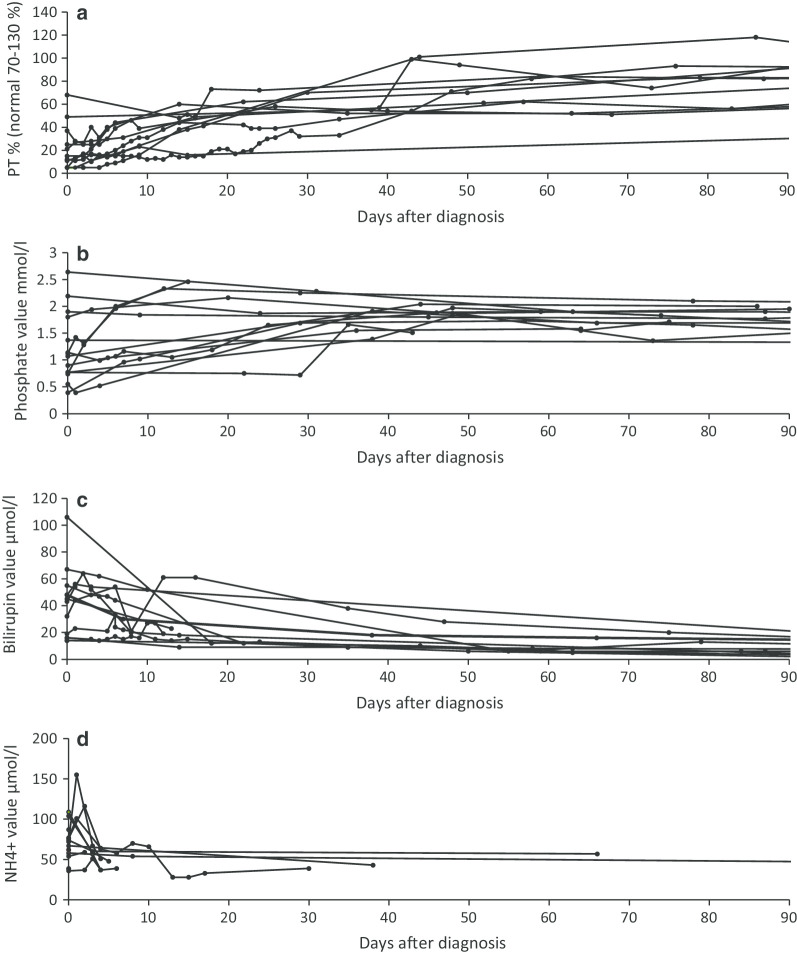
Changes in individual prothrombin time (**a**) and phosphate (**b**), total bilirubin (**c**) and ammonium ion (**d**) values in nitisinone-treated study patients

**Fig. 4:**
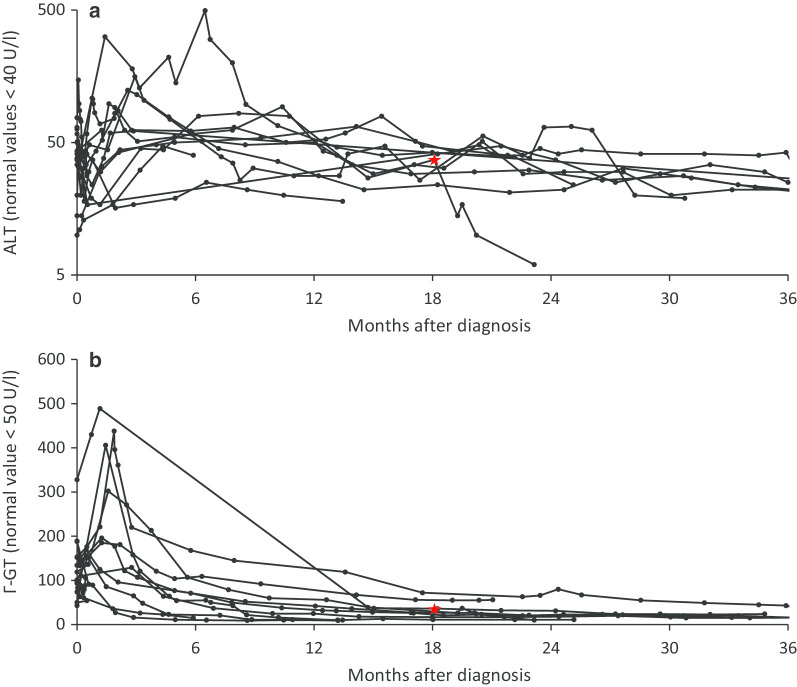
Changes in individual alanine aminotransferase (**a**) and gamma-glutamyl transferase (**b**) values in nitisinone-treated study patients. Red star shows the time of liver transplantation eventually required in one subject

Serum AFP decreased steadily on nitisinone except in one patient with later malignancy (Fig. 2a), while tyrosine and nitisinone levels varied markedly (Fig. 2b, c). In transplanted patients, AFP persisted (Fig. 2a) and tyrosine was kept low until operation (Fig. 2b). Conjugated bilirubin normalized in 10–550 days, albumin in 58–60 days, and haemoglobin in 3–90 days on nitisinone. Thrombocytopenia normalized in 3–90 days, except in three patients, of whom two were later transplanted. All three had persistent or reappearing splenomegaly. Acidosis and tubulopathy disappeared within three months and urine SA within one month. Normalization of the other values is presented in Figs. 3 and 4.

Liver imaging abnormalities persisted until transplantation. On nitisinone, they normalized in 10/13 patients within 56 months; two of the three with persistent findings were later transplanted. Splenomegaly normalized within 56 months except in two transplanted patients and one (diagnosed at 8 months) nitisinone-treated patient. It also appeared later in two transplanted and three nitisinone-treated (diagnosis 5–33 months) patients of whom four developed liver complications. Kidney, central nervous system (CNS) and cardiac findings and rickets normalized within 56 months. However, 5/6 transplanted and 1/6 nitisinone-treated patients with rickets later developed osteopenia/osteoporosis. None of the NBS patients had persistent laboratory or imaging abnormalities and thus none needed transplantation.

### Long-term nitisinone treatment, tyrosine levels and protein intake

The median nitisinone dose (n = 13) was 1.00 (range 0.69–1.83) mg/kg/day and the mean serum level of 212 measurements 56 (12–97) µmol/l. There was no correlation between the levels and dosing or taking the drug once (n = 3) or twice a day (n = 10). Urine SA remained constantly negative even with low nitisinone. Median protein intake during nitisinone was 2.2 (1.0–3.0) and 2.0 (1.3–3.0) mg/kg/day before and after one year of age respectively, and the ratio between natural and tyrosine/phenylalanine-free protein 0.7 (0.2–1.1) and 0.8 (0.3–2.0). Higher natural and modified protein ratio increased the likelihood of later transplantation (p = 0.007).

Low serum nitisinone was associated with later complications, these being present in six patients with mean value ≤ 54 µmol/l and none with > 54 µmol/l (AUC 0.85 [0.63–1.00]; p = 0.040). This was seen particularly with growth failure (0.93 [0.78–1.00], p = 0.028) and later transplantation (0.93 [0.78–1.00], p = 0.028). Also, low minimum nitisinone was associated with learning difficulties (0.86 [0.64−1.00], p = 0.045); three out of four children with minimum ≤ 24 µmol/l and 1/9 of those with > 24 µmol/l). Neither the type nor the number of complications was associated with tyrosine levels or variability of those levels.

## Discussion

We found the incidence of TT1 to be 1/90,102 in Finland, with a significant enrichment (1/9,990) in South Ostrobothnia. The latter is likely due to the homogenous population and overrepresentation of inherited diseases in this area [18]. In central Europe the corresponding figure is ~ 1/100,000–200,000 [6, 19], in Norway 1/74,800 [7] and in Quebec (Canada) up to 1/16,000 [4], whereas in Japan TT1 is exceptional [1]. As a further supporting founder effect, most patients living in South Ostrobothnia had homozygotic “Finnish type” Trp262X mutation [5] and their ancestors also originated from there. The other mutations were c.1062 + 5G > , which is common in Quebec [16, 20], c.191delA common in Turkey [21] and “Mediterranean” c.554-1G > T [20]. Of note, one patient had a previously unreported c.205del (p.Ser69fs) mutation [20].

Clinical presentations were mostly in line with earlier reports [6, 16, 17, 19, 22–27], the main findings including e.g. liver failure, poor growth and rickets. Pregnancies had also been uneventful [17, 23], but quite many had temporary hypoglycaemia or hypotonia after birth and five presented with inguinal/scrotum hernias, likely due to ascites [28]. These previously unreported findings should be kept in mind as possible early signs of TT1 in high-prevalence areas. Inexplicably, we found no cases with frequently described [16, 19, 22, 29] cardiomyopathy and neurological crises. Of note, although according to laboratory parameters most of the patients had deep coagulopathy typical for TT1 [22, 24, 29], severe bleeding was rare.

The number of screen-detected patients was low, but their milder phenotype compared with those detected clinically was evident. Moreover, in line with earlier short-term studies [6, 13, 22, 29], early detection seemed to improve the prognosis; all subjects needing transplantation despite nitisinone had late diagnosis. As regards the benefits of NBS, the current evidence is again based mainly on short-term reports [6, 17, 29], an exception being a Canadian study which found none of the NBS patients to have significant liver problems after 5–10 years [16]. However, like some other groups [6, 17, 29] we observed signs of hepatic dysfunction which, together with the aforesaid neonatal symptoms, suggests disease progression already in utero [17, 30]. The frequency of neonatal hypoglycaemia was also surprisingly high. Although this could be due in part to the sensitive screening performed frequently on Finnish newborns, it may also be TT1-related, and further studies on this interesting issue are needed. Risk of later health problems emphasizes the need for careful follow-up also for screen-detected patients [6].

Basic laboratory values and liver function tests normalized rapidly in most cases, while this took longer in case of transaminases and biliary parameters. This is mostly in line with earlier reports [17, 19, 23, 25], although the follow-up time has usually been shorter. Transaminase levels were higher and normalized more slowly than described before [16, 23], but this did not predict later complications. Some children also presented with high ammonium ion (NH4 +), this being feared to predict early need for transplantation [11], but the values decreased promptly on nitisinone. Slow decline of AFP is a physiological phenomenon in infancy [30, 31], but our findings confirm that nondecreasing or rerising values predict malignancy [16, 19, 22, 32]. Of note, persistent thrombocytopenia, which was associated with splenomegaly and is a classical sign of a chronic liver disease, was also a strong predictor of subsequent need for transplantation.

The kidney, CNS, cardiac and bone imaging findings normalized within a few years, but some patients had persistent liver abnormalities and/or splenomegaly. Comparable gradual improvement in renal findings has also been reported by a British group [25] whereas clinically important data about the disappearance of the other findings or significance of their perseverance has been limited. Persistent liver abnormalities have been reported in 19–57% of patients, but the follow-up times have been shorter than in the present study [6, 16, 17, 19, 23, 29]. Here the nonresponsive findings and reappearance of splenomegaly were major warning signs for subsequent hepatic transplantation.

Long-term complications of TT1 were mostly analogous with those reported in the literature [15–17, 19, 25, 29, 33, 34]. Neurological problems and poor growth were more common here, but the follow-up times of these earlier studies may have been too short to detect these late-appearing issues. The former have been suggested to be partially attributable to the side effects of nitisinone [34, 35], but this is debatable [33, 36] and here low levels seemed more harmful. However, early disease onset seems to increase the risk for neurological complications [6], again supporting the idea of in utero progression of TT1. Poor growth has been sparsely reported^28^, but the possible risk for short stature observed here calls for further studies. The association observed between low mean nitisinone and growth failure may be due to inadequate treatment and ongoing liver disease. Then again, these same patients needed liver transplant despite nitisinone use, and hence the role of transplantation must also be considered. There is also limited and inconsistent data about the prevalence and appropriate follow-up of bone issues in TT1 [12, 16], but our results suggest that surveillance at least for cases with rickets at diagnosis is warranted. In contrast, as in prior short-term studies [17, 19, 23, 25, 29], persistent renal involvement seems to be rare.

We found no significant association between long-term complications and tyrosine levels, but low serum nitisinone, even with negative urine SA, was associated with learning difficulties, growth delay and a need for transplantation. The reported nitisinone target range has varied 20–80 µmol/l [6, 11, 37] and the dosing usually aims to achieve negative urine SA [6, 24]. Optimal levels remain somewhat unclear, but frequent monitoring of the levels with a target of 40–60 µmol/l has been recommended [12]. Interestingly, recent studies have reported increased blood SA with nitisinone concentrations < 44.3 µmol/l [38] and positive urine SA with nitisinone < 40 µmol/l, respectively [39]. Thus, there may in theory have been intermittent SA secretion in our patients with low mean nitisinone levels even without detectable urine SA at follow-up visits. Urine SA varies depending on urine concentration and blood measurement may be more stable and replicable indicator of ongoing SA production [40]. Thus, the latter in combination with the nitisinone level could thus be preferable as a follow-up marker [38–40]. Interpretation of the markedly fluctuating nitisinone values can be difficult, but our results underline the importance of sufficient levels, which may be even higher than previously suggested. Of note, the increased ratio of natural to modified protein was also associated with subsequent need for liver transplantation in nitisinone-treated patients. This may be related to generally higher tyrosine levels, although we found no statistically significant association between tyrosine levels and complications.

The main strengths of the present study were the nationwide coverage and availability of comprehensive medical data. Detailed registers also provided information on long-term outcomes and enabled us to assess risk factors for later complications, although weaknesses in the retrospective design remain. As a further limitation, an intensified register search was conducted in only one district, which in theory could have led to a few earlier cases being missed. Some patients may also have died before the transplantation era and were thus lost since medical records are deleted 12 years posthumously.

## Conclusions

The overall prognosis of TT1 has improved in the nitisinone era and NBS seems to provide further benefits. However, clinicians should realize that a risk of complications persist even among screen-detected patients. Intensified surveillance is warranted, especially in patients with delayed diagnosis and persistent laboratory or imaging abnormalities. In addition, maintaining sufficient nitisinone levels is important.

## Methods

### Study design and patients

Treatment of metabolic disorders in Finland is centralized in the university hospitals of Tampere (TAYS), Helsinki, Turku, Oulu and Kuopio. Patients with TT1 were searched from these centres by contacting the physicians responsible and by applying International Classification of Diseases code E70.2. In addition, since TAYS was known to diagnose most of the TT1 patients due its location near South Ostrobothnia, an intensified search among children with metabolic disease or liver failure diagnosed since 1960 was conducted there. Some follow-up visits took place in the central hospitals of Seinäjoki and Satakunta, and this data was also obtained. After patient identification, comprehensive medical data on each case was collected from birth until Sept. 2019.

### Clinical characteristics and family background

The date of diagnosis was defined as the first positive urine SA, also in the case of NBS performed initially from dried blood spot. TT1 became part of the Finnish NBS programme in 2014 and currently 97% of newborns are tested. Positive blood spot screening result is confirmed by urinary SA measurement. If SA is negative, false positive NBS is confirmed by measuring plasma amino acids and urine organic acids [12, 41]. The collected data comprised a diagnostic approach (clinical suspicion vs. screening), demographic data, symptoms and clinical findings, developmental stage and growth parameters [42, 43] and relevant data about pregnancy and delivery. In addition, the origins of patients’ grandparents or the birthplace of the patient, presence of other TT1 cases in the family and possible consanguinity were documented.

### Laboratory and imaging findings

The collected blood values included alanine aminotransferase (ALT), albumin, ALP, AFP, NH4+, blood count, calcium, γ-GT, glucose, PT and total (TBil) and conjugated bilirubin. The presence of liver or kidney dysfunction, kidney tubulopathy, anaemia, thrombocytopenia and hypoglycaemia [11, 26, 44–46] were also recorded. Liver dysfunction was diagnosed by the clinician based on the presence of characteristic findings (e.g. coagulopathy, hypoglycaemia, hypoalbuminemia, low cholesterol, increased NH4^+^). Kidney tubulopathy was based on the presence of acidosis and abnormal urine protein and/or glucose and/or microglobulin. During nitisinone treatment, the above-mentioned laboratory values, blood tyrosine and nitisinone levels and urine SA were monitored until current date. In transplanted patients, AFP and tyrosine values were monitored until transplantation.

The presence of ascites, abnormalities of liver, spleen, kidney, heart and CNS and the presence of rickets and osteopenia/osteoporosis were evaluated using X-rays, ultrasound, computer tomography, magnetic resonance and bone densitometry. Besides diagnostic imaging, at least annual abdominal surveillance was conducted in all patients. The disappearance of the abnormalities and the appearance of new findings were also documented.

### Treatment

Possible treatment modalities included sole dietary restriction, liver transplantation and nitisinone. Histology of the explants and possible transplantation complications were recorded, as were the compliance to and dosing (mg/kg/day) of nitisinone.

### Long-term outcomes

Possible later complications were categorized to surgical complications, need for retransplant or transplantation despite nitisinone, kidney dysfunction, hypertension, pseudo-porphyric crises [11, 12, 47], osteopenia/osteoporosis, cardiac and ophthalmologic complications, delayed growth and neurological problems [11]. Kidney dysfunction was defined as decreased glomerular filtration rate. Cause of death were also noted.

### Statistical analysis

Categorical variables are reported as numbers and percentages, and numerical data as medians with quartiles or ranges. Comparisons were made with Mann–Whitney test, Chi-square or Fisher’s exact test as appropriate. Associations between diagnostic findings and later complications were analysed with Spearman’s correlation and binary logistic regression, which was used for age adjustments, and those between nitisinone and tyrosine levels and complications with ROC [48] and crosstabulation. The possible association between low minimum nitisinone level (the lowest level of an individual patient during treatment) and later complications was also tested for each patient. Levels measured at the beginning of treatment while still adjusting the dosage were excluded. Similarly, the association between mean nitisinone and tyrosine levels (all values measured during follow-up visits) and risk for later complications was assessed. Specifically, ROC curve and crosstabulation were used to find the nitisinone and tyrosine levels at which the risk of later complications started to increase. Incidence was the number of TT1 patients divided with all live births. General birth data was provided by Statistics Finland [49]. Coefficient of variation (SD/mean) illustrated variability of blood tyrosine and nitisinone concentrations in each patient. Delayed diagnosis was considered to be a delay longer than the median of the study patients. Significance was defined as P value < 0.05. Analyses were performed using SPSS Statistics 25.0. (IBM Corp Armonk, NY).

## Data Availability

All data generated or analysed during this study are included in this published article.

## Abbreviations

AFP: Alpha-fetoprotein
ALT: Alanine aminotransferase
ALP: Alkaline phosphatase
AUC: Area under the curve
CNS: Central nervous system
γ-GT: Gamma-glutamyl transferase
NBS: Newborn screen
NH4+: Ammonium ion
PT: Prothrombin time
SA: Succinylacetone
SD: Standard deviation
TAYS: Tampere University Hospital
TBil: Total bilirubin
TT1: Type 1 tyrosinemia
